# Projected number of people in need for long-term care in Germany until 2050

**DOI:** 10.3389/fpubh.2024.1456320

**Published:** 2024-10-30

**Authors:** Luisa Haß, Stephanie Knippschild, Thaddäus Tönnies, Annika Hoyer, Rebecca Palm, Sabrina Voß, Ralph Brinks

**Affiliations:** ^1^Faculty of Health/School of Medicine, Chair for Medical Biometry and Epidemiology, Witten/Herdecke University, Witten, Germany; ^2^Institute for Biometrics and Epidemiology, German Diabetes Center (DDZ), Leibniz Center for Diabetes Research at Heinrich Heine University, Düsseldorf, Germany; ^3^Biostatistics and Medical Biometry, Medical School OWL, Bielefeld University, Bielefeld, Germany; ^4^Department of Health Service Research, Carl von Ossietzky Universität Oldenburg, Oldenburg, Germany; ^5^Faculty of Health, School of Nursing Science, Witten/Herdecke University, Witten, Germany

**Keywords:** epidemiology, chronic condition, aging, illness-death model, long-term care, partial differential equation, aggregated data

## Abstract

**Introduction:**

Current demographic trends predict continuously growing numbers of individuals reliant on care, which has to be accounted for in future planning of long-term care-resources. The projection of developments becomes especially necessary in order to enable healthcare systems to cope with this future burden and to implement suitable strategies to deal with the demand of long-term care. This study aimed to project the prevalence of long-term care and the number of care-dependent people in Germany until 2050.

**Methods:**

We used the illness-death model to project the future prevalence of long-term care in Germany until 2050 considering eight different scenarios. Therefore, transition rates (incidence rate and mortality rates) describing the illness-death model are needed, which have been studied recently. Absolute numbers of people in need for long-term care were calculated based to the 15th population projection of the Federal Statistical Office.

**Results:**

Numbers of people in need for long-term care will increase by at least 12%, namely 5.6 million people, in the period of 2021 until 2050. Assuming an annual incidence-increase of 2% from 2021 to 2050 the number of care-dependent individuals could potentially rise up to 14 million (+180%).

**Conclusion:**

Our projections indicated a substantial rise in the number of care-dependent individuals. This is expected to lead to raising economic challenges as well as a stronger demand for healthcare and nursing personnel.

## 1 Introduction

Germany is the most populous country within the European Union and plays an enormous economic and social role (1). In 2022, more than a third of the German population was aged 65 years and above with a clear upward trend from the years before (2), which raises the question how the number of people in need for long term-care (LTC) will develop. Decision makers in the government made urgent responses in legislation: The German “Pflegestärkungsgesetz I-III” (PSG I-III; 2015–2017) assigned the task of coordinating the area of care in order to create a suitable, sufficient and economically viable care infrastructure to the federal states of Germany. The states have issued regulations obliging the municipalities to regularly draw up an inventory of care services, identify gaps or oversupplies in care, and formulate measures to meet demand [§§ 8a, 9 Social Security Code Book XI (SGB XI)] (3).

The current demographic shifts associated with population aging (4, 5) indicate a continuous increase in the number of care-dependent individuals in Germany (4), as the need for LTC depends on age. Recent studies indicate that approximately 5 million individuals in Germany require and receive LTC (4, 5). As life expectancy continues to increase, particularly in conjunction with the aging of the baby boomer generation, the already strained care situation (6, 7) will become increasingly affected. This is associated with challenges such as the staffing situation in care facilities and the financial burden due to the out-of-pocket expenses patients have in addition to what LTC insurance covers (co-payments) for LTC (4). However, the political framework conditions, in particular with regard to the number of care-dependent individuals (recipients) and the care structure, are also important for the further development of LTC insurance policies (5, 8).

Due to these current and expected developments, a projection of the number of individuals in care-dependency^1^ in Germany is of great importance in order to enable targeted resource planning, the establishment of demand-oriented care structures and the promotion of preventive measures to improve the quality of life for people in need for LTC. While existing studies provide only prevalence-based trends of LTC needs, our analysis aims to project the future prevalence and number of people in need for long-term care in Germany until 2050 using an illness-death model.

## 2 Methods

### 2.1 Definition of long-term care

As defined by the SGB XI, care-dependent individuals are characterized as those who exhibit health-related impairments in their autonomy or capabilities to fulfill daily routines, necessitating assistance from others. These individuals are unable to independently compensate for or manage physical, cognitive, or psychological impairments, as well as health-related burdens or demands. The condition of care-dependency must persist for a duration of at least 6 months, with a severity level meeting the minimum criteria specified in § 15 (§ 14 Abs. 1 SGB XI) (5).

### 2.2 Database

We retrieved prevalence data (Table 1.2 (5): “Pflegequote”) about the need for LTC in Germany of 2021 from official statistics published by the German Federal Statistical Office (FSO), comprising 84 million people (5). These data were collected as part of the national law in Germany and capture all individuals receiving benefits according to SGB XI. The fundamental requirement for inclusion is the determination of care-dependency and the allocation to care grades 1–5 by the national statutory LTC insurance or a private insurance company.

Moreover, the 15th coordinated population projection of the FSO (9) was used for this projection and final calculation of the number of people in need for LTC. In accordance with the development of the German population, these data are available for different scenarios (different assumptions on birth rate, life expectancy and migration patterns). We used variants 1 and 2 from the German FSO (9), which account for historical trends in population growth and provide a conservative basis. Both variants assume a birth rate of 1.55 children per woman and a long-term net migration of 250,000 people per year. Variant 1 projects a life expectancy at birth in 2070 of 82.6 years for boys and 86.1 years for girls, while variant 2 projects a life expectancy of 84.4 years for boys and 88.1 years for girls (9).

Furthermore, all-cause mortality, mortality rate ratio, and incidence rate are required. The all-cause mortality data is derived from the German FSO (10). Given the absence of data for the mortality rate ratio in Germany, a systematic literature search in (11) identified a range of 1.17–3.2. Therefore, both values need to be considered in the scenarios presented. Additionally, incidence rates [scenario 1 and 2 (Supplementary Table S1)] from Haß et al. (11) were used.

### 2.3 Illness-death model and partial differential equation

**Figure 1:**
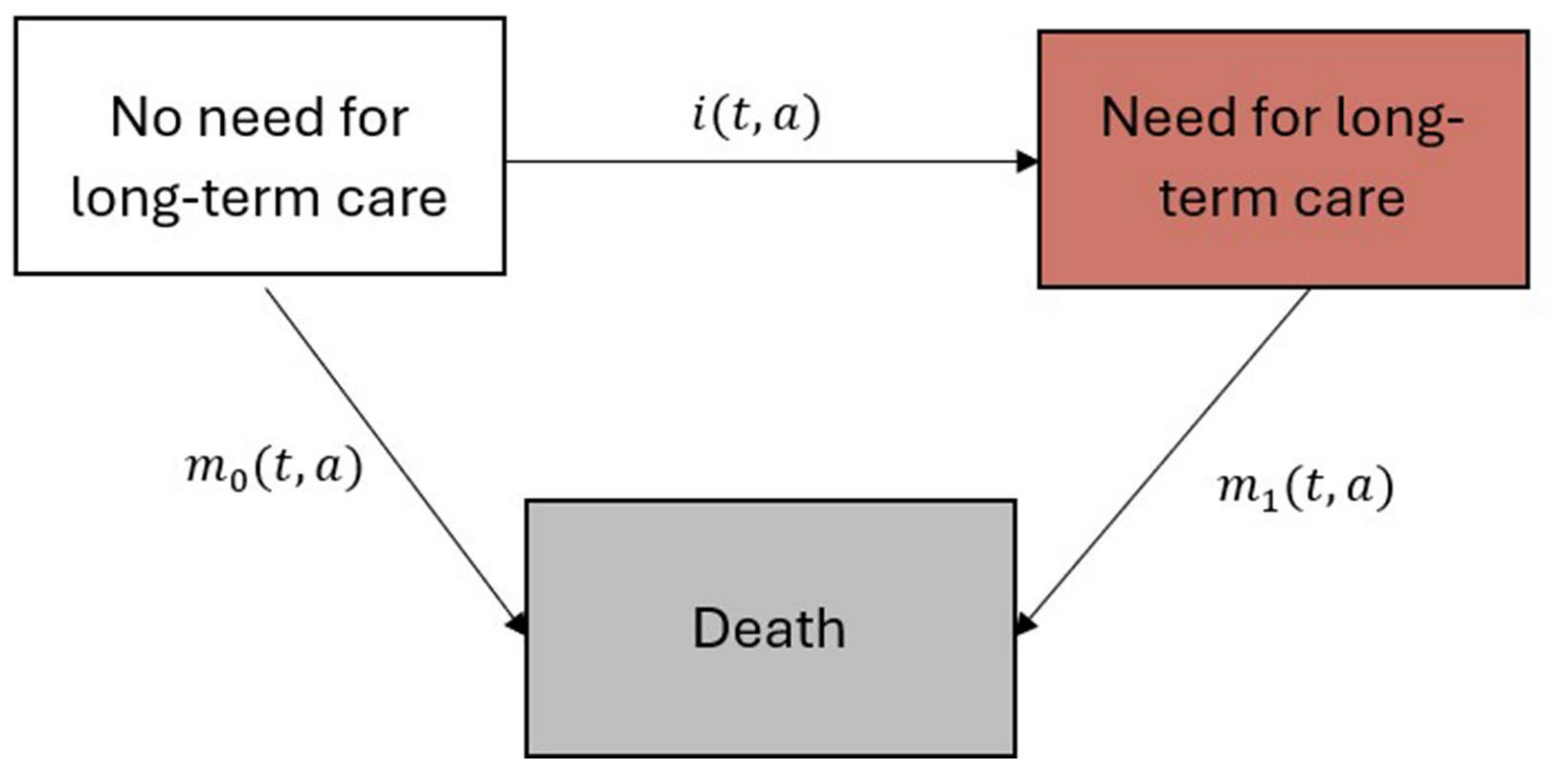
Illness-death model with the three states “No need for long-term care,” “Need for long-term care” and “Death.” Age- *(a)* and calendar time *(t)* dependent incidence rate of need for long-term care *(i)* and the mortality rates for individuals without a need for long-term care *(m_0_)* and in need for long-term care *(m_1_)* describe the underlying dynamics of the model (12–14).

This study projects the prevalence and number of people needing LTC in Germany until 2050 using an illness-death model (Figure 1) and a related partial differential equation (PDE), which account for the dynamic nature of health transitions by considering the relationships between prevalence, mortality rates, and incidence rates of need for LTC as a chronic condition (12–14).

The model includes three distinct states: “No need for long-term care,” “Need for long-term care,” and “Death.” The incidence rate of need for long-term care (*i*), the mortality rates for individuals without need for LTC (*m_0_*) and with need for LTC (*m_1_*) describe the age- (*a*) and calendar time (*t*) dependent transitions between the states (Figure 1). This illness-death model is governed by a PDE, which provides a mathematical framework for describing the temporal changes in age-and calendar time dependent prevalence as a function of the transition rates between the states (12–15).

We used the all-cause mortality rate *(m)* for Germany, the prevalence *(p)*, the incidence rate of LTC *(i)*, and the mortality rate ratio *(R)* - which relates the mortality rates between two groups (*m_1_*/*m_0_*) - to solve the PDE to calculate the annual prevalence of LTC from 2021 to 2050. For the calculation and projection of future prevalences, the following PDE was solved with Runge–Kutta-fourth-order method (16) implemented in R-package deSolve (17):


$$\left(\frac{\partial }{\partial t}+\frac{\partial }{\partial a}\right)p=\left(1-p\right)\left\{i-m\frac{p\left(R-1\right)}{1+p\left(R-1\right)}\right\}$$

### 2.4 Statistical analysis

The initial prevalence of 2021 was set to the prevalence of need for LTC in the group aged 60 to 90+ years (Table 1.2 (5): “Pflegequote”). The analysis was conducted for ages ranging from 60 to 100 years with one-year steps and covered a time span of 30 years in calendar-time (2021–2050).

The solution of the PDE was calculated separately for the two population projection variants from the German FSO (9), the different mortality rate ratios (Supplementary Table S1) and sex. Thus, the projection is based on eight different scenarios for the age-and sex-specific prevalence (Table 1). Scenario 1 and scenario 2 contained a mortality rate ratio of *R* = 3.2, as well as the two variants of the population projection for men and women separately. For the third and fourth scenario, however, another mortality rate ratio of *R* = 1.17 was used. Additionally, in scenarios 5 to 8, an annual increase of incidence rates of 2% was included. This 2% increase of the incidence rate is the lower bound for the annual increase based on the trend in the past two decades (18).

**Table 1:** Considered scenarios for projecting people in need for LTC.

Scenario	Population projection variant	Mortality rate ratio	Annual increase in incidence rate
1	1	3.2	
2	2	3.2	
3	1	1.17	
4	2	1.17	
5	1	3.2	2%
6	2	3.2	2%
7	1	1.17	2%
8	2	1.17	2%

To calculate the future number of people in need for LTC, the projected age-and sex-specific prevalences were applied to the projected future age-and sex-specific populations in Germany. Therefore age-and sex-specific LTC prevalences were multiplied with the projected number of individuals in Germany from the German FSO for each year (9) until 2050. A detailed description of this projection method including a comparison with other methods can be found in Voeltz et al. (15).

All calculations were performed with the freely accessible, open-source statistical software R Version 4.2.3 (The R Foundation for Statistical Computing). Data and source codes for the analysis are available in the permanent, freely accessible online repository Zenodo (19).

## 3 Results

The number of people in need for LTC in Germany in 2050 was calculated in eight different scenarios (Table 1). Overall, our findings indicate a persistent and progressive increase in the number of care-dependent individuals in Germany across all scenarios.

**Figure 2:**
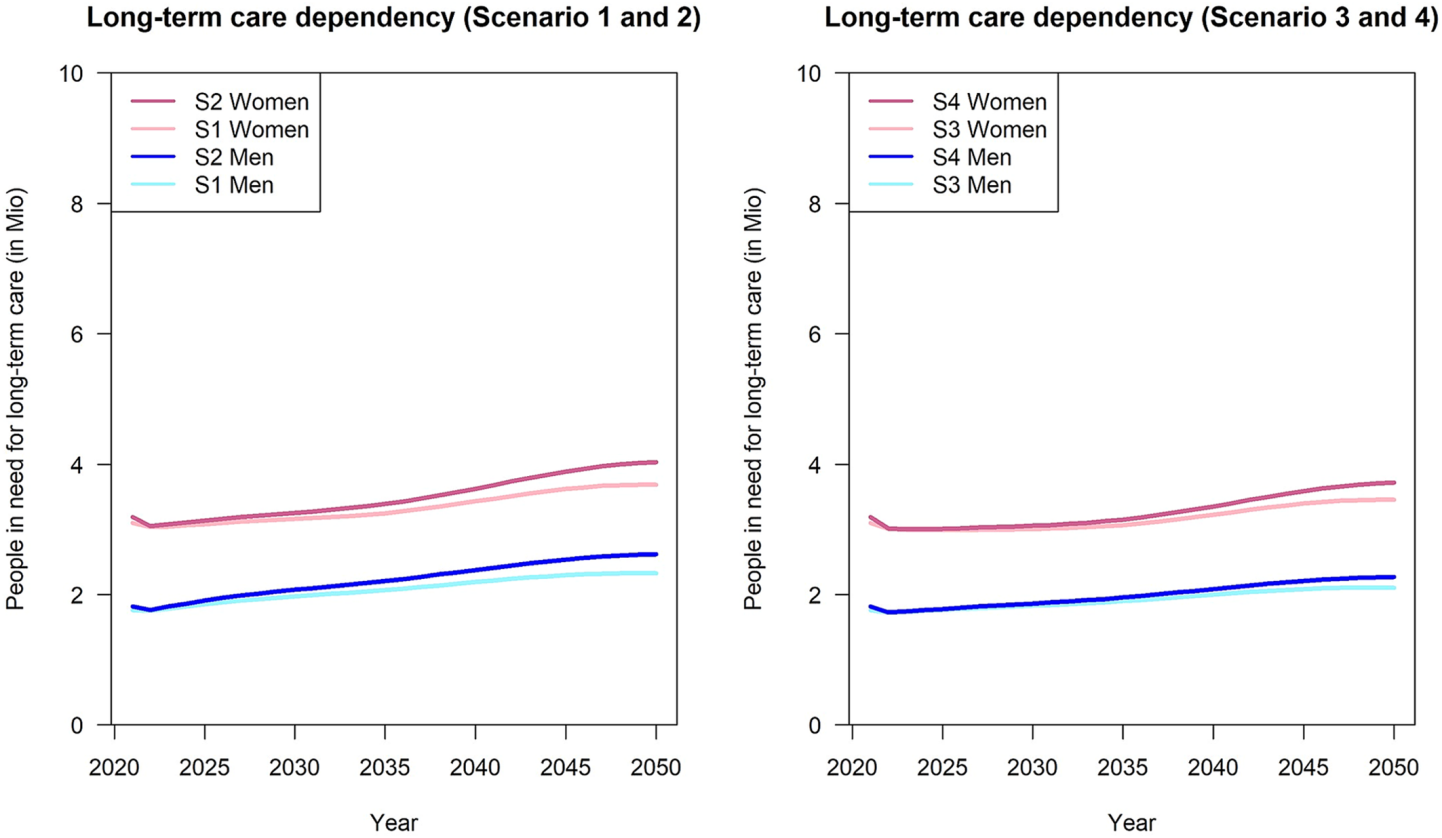
Number of people in need for long-term care from 2021 to 2050, stratified by sex and population projection variant. S1: Variant 1, *R* = 3.2; S2: Variant 2; *R* = 3.2, S3: Variant 1, *R* = 1.17; S4: Variant 2, *R* = 1.17.

In 2021, an approximate total of 5 million individuals needing LTC in Germany as defined by the German national law (SGB XI) was recorded, corresponding to a prevalence of 6% (5) of the total German population. The left side of Figure 2 depicts the projected sex-specific number of individuals in need for LTC for the first and second scenario (*R* = 3.2 and both variants of the population projection) from 2021 to 2050. In comparison, scenarios 3 and 4, shown in the plot on the right (Figure 2), demonstrate a less notable increase in the projected number of individuals requiring LTC (Table 2). Among men, Scenario 2 exhibits the greatest percentage change, with an 44% increase from 1.8 million to 2.6 million people in need for LTC (Table 2). In contrast, scenario 3 (women) displays the smallest increase of 9% (Table 2). Overall expected counts of individuals in need for LTC in Germany in 2050 exhibit only marginal discrepancies between the different scenarios. Considering factors like the increasing life expectancy (population projection variant 2) and age-specific incidence rates (Supplementary Table S1) under the assumption of a constantly high mortality rate ratio (*R* = 3.2), the projected cases are expected to increase by 1.6 million individuals (+32%) (Table 2: Scenario 2).

**Table 2:** Number of people in need for long-term care in eight scenarios for 2021 and 2050 by sex, mortality rate ratios and population projection.

Scenario	2021 (in Mio.)			2050 (in Mio.)		
	Men	Women	Total	Men	Women	Total
1	1.8	3.2	5.0	2.3 (+28%)	3.7 (+16%)	6.0 (+20%)
2	1.8	3.2	5.0	2.6 (+44%)	4.0 (+25%)	6.6 (+32%)
3	1.8	3.2	5.0	2.1 (+17%)	3.5 (+9%)	5.6 (+12%)
4	1.8	3.2	5.0	2.3 (+28%)	3.7 (+16%)	6.0 (+20%)
5	1.8	3.2	5.0	5.6 (+211%)	7.6 (+138%)	13.2 (+164%)
6	1.8	3.2	5.0	6.0 (+233%)	8.0 (+150%)	14.0 (+180%)
7	1.8	3.2	5.0	5.2 (+189%)	7.3 (+128%)	12.5 (+150%)
8	1.8	3.2	5.0	5.6 (+211%)	7.7 (+141%)	13.3 (+166%)

**Figure 3:**
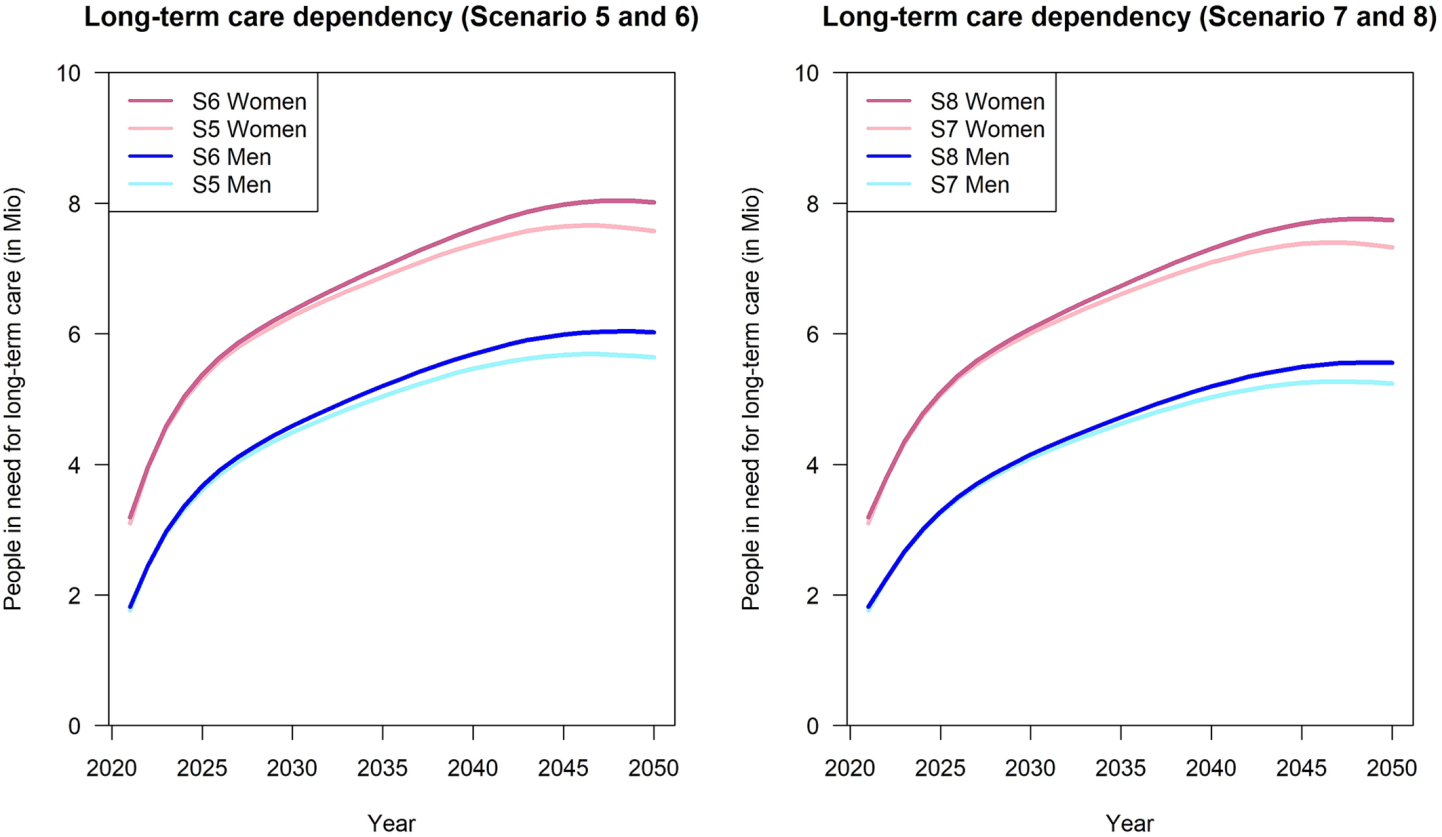
Number of people in need for long-term care from 2021 to 2050, stratified by sex and population projection variant. S5: Variant 1, *R* = 3.2, 2% annual increase; S6: Variant 2, *R* = 3.2, 2% annual increase; S7: Variant 1, *R* = 1.17, 2% annual increase; S8: Variant 2, *R* = 1.17, 2% annual increase.

Assuming an annual increase in the incidence rate of 2% for both scenarios, the number of care-dependent individuals will have at least doubled by the year 2050 (Figure 3; Table 2: scenarios 5–8). For instance, scenario 3 (Variant 1, *R* = 1.17, 2% annual increase in incidence), which shows the lowest increase among scenarios assuming an annual increase in incidence, approximately 12.5 million people in need for LTC (+150%) are estimated. Moreover, as mentioned before, there are also only marginal differences across the different scenarios.

## 4 Discussion

### 4.1 Summary

To our knowledge, this analysis was the first to project the number of people in need for LTC until 2050 in Germany under consideration of the relationship between prevalence, mortality and incidence rate described by an illness-death model. The 15th coordinated population projection (9) and the official LTC statistics published by the German FSO (5) served as the basis. In addition, all-cause mortality (10), mortality rate ratios and the incidence rate of LTC in Germany were integrated (11). To account for uncertainties regarding future trends, we considered multiple scenarios for projecting future prevalence and the calculation of future numbers of people in need for LTC by multiplying calculated future prevalence with projected future population sizes. Consequently, our sex-and age-specific analysis accounted for variations in prevalence, incidence rate, and mortality rate ratio as well as projected demographic shifts until 2050 and comprised the data of 84 million people.

The results show that a minimum number of 5.6 million people in need for LTC will challenge the German healthcare system until 2050, assuming a mortality rate ratio of *R* = 1.17 and variant 1 for the projected population. However, under the assumption of a higher mortality rate ratio (*R* = 3.2), the projected number of care-dependent individuals is expected to rise by more than 20 percentage points. Considering an annual increase in incidence rates for both scenarios, the number of people in need for LTC will at least more than double to a minimum number of 12.5 million.

### 4.2 Comparison to the projection by the German Federal Statistical Office

Besides our projection of future numbers of people in need for LTC other solely prevalence-based projections with a similar data basis of the expected burden of need for care exist. As an example, the prevalence-based projection by the German FSO (20) expects the number of care-dependent individuals in Germany to increase by 34% by 2050 due to the aging population. Their projected number will rise from approximately 5 million at the end of 2021 to about 6.7 million in 2050 (variant 1 (20)). Considering temporarily increasing prevalence until 2027 and from 2027 constant prevalence (variant 2 (20)), the number of people in need for LTC is projected to rise up to 7.5 million in 2050 (+50%). As stated, the increase will primarily be driven by individuals aged 80 years and above (20). When considering the same number of individuals affected by the end of 2021, our results indicate a minimum increase to 5.6 million (Table 2: Scenario 3), 6.6 million (Table 2: Scenario 2) or nearly three times as many, depending on the scenario (Table 2: Scenarios 5–8) (Supplementary Table S2).

In contrast to the prevalence-based calculation by the German FSO, assuming no or just some changes in current LTC conditions (20), our projection differs in assuming constant incidence rates as well as annually increasing incidence rates. This seems plausible, since it is almost impossible for the prevalence to remain constant over time, as there will always be changes in mortality. Hence, if mortality changes in future, the incidence rate would have to increase or decrease at the same rate to ensure constant prevalence.

### 4.3 Limitations

It is important to consider certain limitations when interpreting our results.

The execution of the PSG I-III obliges local authorities to engage in prospective planning and quantification of individuals requiring LTC. Unfortunately, due to the utilization of aggregated federal-level databases in this study, we are unable to provide data at the municipal level, which is equally essential. Consequently, comprehensive and more refined data are needed to effectively address the demands of the (local) healthcare system.

Additionally, in 2017 a new concept of care-dependency has been implemented, encompassing both physical and psychological impairments. In cases where data still contained entries based on the previous care levels (I-III) and classifications as individuals with restricted everyday competence, they were re-coded into care grades (1–5) according to the transitional key outlined in § 140 SGB XI (5). This change provides a more comprehensive representation of the population in need for care but may impact the comparability of data over time and our projection. Additionally, it is important to acknowledge changes in data collection methods by the FSO, which included the first-time registration of individuals with care grade 1, as well as those without a care level but with significantly limited everyday skills. Moreover, the incorporation of data from outpatient care services, which were not considered previously, further contributed to an increase in the number of people identified as in need for care (according to § 71 paragraph 1a SGB XI). These ongoing adjustments in the system may lead to a system-related increase in the prevalence of LTC needs, potentially affecting the accuracy of this calculation.

It should also be noted that the enormous range of the results between scenarios is due to the added annual increase in incidence. However, as described in Voß et al. (2023), the annual increase of 2% is a lower bound estimate of the increase in incidence, which can vary greatly depending on age group and sex (18). In this respect, our findings are a conservative lower estimate.

Apart from the mentioned data limitations, further uncertainties arise due to insufficient information in Germany, specifically the absence of the actual mortality rate ratio (*R*) required for age-sex-specific LTC need incidence rate calculation. This resulted in a range of values (lower and upper bound) derived from the literature search conducted in Haß et al. (11). Due to the lack of data on the mortality rate ratios, constant mortality rate ratios were employed in this calculation. However, these may be influenced both positively and negatively over time by factors such as medical advancements and/or the increasingly severe shortage of skilled workers. Consequently, all identified trends should be regarded as potential tendencies. Furthermore, as already mentioned in Haß et al. (11) the remission rate used to calculate the incidence rate was assumed to be zero. Generally, depending on existing comorbidities, individuals in need for care may potentially regain independence. However, remission is less common than care termination due to death (8). Consequently, the calculated incidence rate serves as the “lower limit” for the risk of requiring care. Bridging these gaps, however, requires studies on aging and LTC with transparent results that incorporate all-cause mortality, varying mortality rate ratios as well as remission rates for different diseases.

Nevertheless, in 2019, approximately 36% of individuals requiring care were provided for in either stationary care facilities or by outpatient services, leading to the involvement of approximately 1.6 million caregivers (21). This signifies a care ratio of approximately 1:1.11. Should the number of caregivers remain constant, decrease, or only slightly increase, this ratio could be expected to worsen dramatically. In the most optimistic scenario (Table 2: Scenario 3), with a stable percentage of stationary and outpatient services for care recipients, the ratio could increase to approximately 1:1.24. However, in the least optimistic scenarios (Table 2: Scenarios 5–8), it could rise significantly to as high as 1:3.1, where one caregiver would be responsible for the care of up to three individuals. It is important to note that these estimates do not account for potential regional disparities, which may further exacerbate the issue in certain areas.

Further data analyses, including those focusing on workforce projections and financial resource requirements, could enhance the research on the growing necessity for LTC. Such analyses would enable a more precise estimation of future demand for skilled personnel and the potential deficit in the caregiving workforce. Furthermore, integrating data on financial resources and funding structures would be beneficial for evaluating the capacity of the current system to accommodate the rising costs of care provision. Additionally, analyzing regional disparities in care needs and workforce availability could provide more practically useful insights for local policymakers.

Although this paper does not aim to provide specific, evidence-based recommendations, it is reasonable to suggest that certain strategies could be explored in order to mitigate future challenges. For example, one potential strategy could involve the establishment of regional LTC task forces that monitor and respond to fluctuations in local demand, thereby ensuring a more efficient distribution of resources and personnel. The introduction of more precise policies designed to motivate young professionals to pursue careers in the care sector and a systematic reform of the field could prove an effective strategy for addressing the critical shortage of workers in the care sector. The integration of technology into home care services, including the use of telemedicine and AI-assisted care, may also help alleviate the burden on especially informal caregivers (family members). These suggestions represent a starting point for further investigation and are not intended to be definitive solutions. Given the complexity of the issue, it is essential to conduct a comprehensive and nuanced analysis by experts in gerontology, health economics, and labor market dynamics to develop effective and actionable strategies.

However, in contrast to these limitations, our analysis has a great advantage: Rather than merely relying on the age-and sex-specific prevalence data from a base year, our projection is based on an illness-death model and incorporates data on various LTC-specific information, including the incidence rate and mortality rate ratio of individuals requiring LTC. Thus, a prevalence-based projection may be insufficient to accurately reflect the complexities of reality (21). Although the IDM has not yet been employed for LTC projections, it is likely to be imitated internationally due to its relatively high degree of accuracy (22). Therefore, we conducted a comprehensive projection of estimated future prevalences and numbers of care-dependent individuals until the year 2050.

## 5 Conclusion

Our projection of future numbers of people in need for LTC in Germany up to 2050 is the first analysis that is based on the relationship of prevalence, incidence rate and mortality of a chronic condition that is described by the illness-death model. The number of affected individuals will at least increase to 5.6 million in 2050 and assuming an annual increase in the incidence rate of 2% at worst even up to 14 million. Irrespective of the scenario, all projections consistently demonstrated an increase in the number of individuals in need for LTC between 2021 and 2050. It is important to note that applying current age-specific prevalence estimates to projected future age structures likely underestimates the magnitude of this increase.

However, due to limited data on the epidemiology of LTC in Germany, it has only been possible to perform calculations of trends on the number of people in need for LTC.

In conclusion, our projection of the future demand for LTC in Germany up to 2050 provides critical insights into the evolving care situation, especially considering arising expected economic challenges and an even greater demand for healthcare and nursing personnel. By acting on recommendations, such as enhanced data collection, strategic resource allocation, healthcare workforce development and collaborative policy initiatives, Germany can better prepare itself to provide sustainable LTC services that align with the evolving needs of its aging populations.

## Data Availability

Availability of data and materials. Publicly available datasets were analyzed in this study. The underlying prevalence data can be accessed from the “Pflegestatistik - Pflege im Rahmen der Pflegeversicherung Deutschlandergebnisse” from the year 2021 (see literature link included in the text or https://www.statistischebibliothek.de/mir/receive/DESerie_mods_00000940). Age- and sex-specific prevalence data were extracted from Tab. 1.2 in reference (5) for women and men. Mortality data were obtained from the official homepage of the Federal Statistical Office: https://service.destatis.de/bevoelkerungspyramide/ with the assumption of a moderate development of birth rate, life expectancy and migration (G2L2W2) as well as assumption of a moderate development of birth rate, migration and lower development of life expectancy (G2L1W2). Incidence rates were derived from our previously published paper: https://f1000research.com/articles/12-102. Definition of sexes within this analysis was used in accordance with the definition in the published data. Source code: Zenodo. https://doi.org/10.5281/zenodo.10715074.

## Footnotes

^1^Need and use will be used synonymously throughout this paper. This is because in Germany, every individual who needs care (is care-dependent) receives some kind of care (financial support, care support, etc.).
