# Supplementary material for: Projected number of people in need for long-term care in Germany until 2050
Source: Front Public Health. 2024 Oct 30;12:1456320. doi: 10.3389/fpubh.2024.1456320 (PMC11558338; doi:10.3389/fpubh.2024.1456320)
Supplement: Supplementary file 1 [file Table_1.docx]

Supplementary Material

Projected number of people in need for long-term care in Germany until 2050

Luisa Haß*, Stephanie Knippschild, Thaddäus Tönnies, Annika Hoyer, Rebecca Palm, Sabrina Voß*, Ralph Brinks

*Correspondence: Luisa Haß: [luisa.hass@uni-wh.de](mailto:luisa.hass@uni-wh.de); Sabrina Tulka: [sabrina.tulka@uni-wh.de](mailto:sabrina.tulka@uni-wh.de)

1 Supplementary Table S1

*Supplementary Table S1: Incidence rate per 1000 Person-Years in two scenarios from 2015 by sex, age and mortality rate ratios (R) [12]. Scenario 1: R = 3.2, scenario 2: R = 1.17 for all ages.*

| **Age (Years)** | **Scenario 1 (*R = 3.2*)** | | **Scenario 2 (*R = 1.17*)** | |
| --- | --- | --- | --- | --- |
|  | **Men** | **Women** | **Men** | **Women** |
| **50** | 1.30 | 1.03 | 1.24 | 1.01 |
| **60** | 3.08 | 2.80 | 2.81 | 2.71 |
| **70** | 9.62 | 10.3 | 7.95 | 9.38 |
| **80** | 38.3 | 47.7 | 27.2 | 39.2 |
| **90** | 153 | 200 | 94.4 | 145 |

2 Supplementary Table S2

*Supplementary Table S2: Comparison between the projected total numbers of people in need of long-term care published by the German Federal Statistical Office [20] and the presented projection. “Status quo”: Constant prevalences, Variant 2: temporarily increasing prevalences until 2027 and from 2027 constant prevalences, S1: Scenario 1 (Variant 1 & R = 3.2) / scenario 2 (Variant 2 & R = 3.2), S2: Scenario 3 (Variant 1 & R = 1.17) / scenario 4 (Variant 2 & R = 1.17), S3: Scenario 5 (Variant 1, R = 3.2, 2% annual increase) / scenario 6 (Variant 2, R = 3.2, 2% annual increase), S4: Scenario 7 (Variant 1, R = 1.17, 2% annual increase) / scenario 8 (Variant 2, R = 1.17, 2% annual increase).*

| **2050 (in Mio.)** | **2050 (in Mio.) + annual increase** | **“Status quo”** | **Variant 2** |
| --- | --- | --- | --- |
| **Total** | **Total** | **Total** | **Total** |
| 6.0 / 6.6 (S1) | 13.2 / 14.0 (S3) | 6.7 | 7.5 |
| 5.6 / 6.0 (S2) | 12.5 / 13.3 (S4) | 6.7 | 7.5 |

3 Availability of data and materials

Publicly available datasets were analyzed in this study.

The underlying prevalence data can be accessed from the “Pflegestatistik - Pflege im Rahmen der Pflegeversicherung Deutschlandergebnisse“ from the year 2021 (see literature link included in the text or<https://www.statistischebibliothek.de/mir/receive/DESerie_mods_00000940> ). Age- and sex-specific prevalence data were extracted from Tab. 1.2 in reference [6] for women and men.

Mortality data were obtained from the official homepage of the Federal Statistical Office:<https://service.destatis.de/bevoelkerungspyramide/> with the assumption of a moderate development of birth rate, life expectancy and migration (G2L2W2) as well as assumption of a moderate development of birth rate, migration and lower development of life expectancy (G2L1W2).

Incidence rates were derived from our previously published paper: <https://f1000research.com/articles/12-102>

Definition of sexes within this analysis was used in accordance with the definition in the published data.

Source code: Zenodo. https://doi.org/10.5281/zenodo.10715074

Figures and Tables: The figures presented within this analysis based on the above-mentioned data, were created by the authors of this article.
